# Unique bifunctional α-sialidase/β-*N*-acetylgalactosaminidase from *Bifidobacterium bifidum* acting on the Sd^a^ antigen

**DOI:** 10.1016/j.jbc.2025.111121

**Published:** 2025-12-30

**Authors:** Toshihiko Katoh, Rina Suzuki, Shogo Kataoka, Junya Kawasaki, Keijiro Kamio, Masahiro Komeno, Saya Yoshioka, Annemette Tengstedt Rasmussen, Ikuo Kimura, Takane Katayama, Hisashi Ashida

**Affiliations:** 1 Division of Integrated Life Science, Graduate School of Biostudies, Kyoto University, Kyoto, Japan; 2 Graduate School of Biology-Oriented Science and Technology, Kindai University, Wakayama, Japan; 3 Division of Systemic Life Science, Graduate School of Biostudies, Kyoto University, Kyoto, Japan

**Keywords:** sialidase, neuraminidase, β-*N*-acetylgalactosaminidase, *Bifidobacterium bifidum*, GH123, GH33, mucin, probiotics

## Abstract

Sd^a^ antigens [GalNAcβ1–4(Neu5Acα2-3)Galβ1-*O*-R] are present at the nonreducing termini of *O*-glycans of colonic mucins of humans. Previously, we reported characterization of two glycoside hydrolase (GH) family 33 α-sialidases, SiaBb1 and SiaBb2, from a symbiotic *Bifidobacterium bifidum* dwelling in the human intestines. In this study, we identified a third α-sialidase, SiaBb3 from *B. bifidum*, that is distinguished from the aforementioned two sialidases by its possession of an additional GH123 β-*N*-acetylgalactosaminidase domain within the same polypeptide. The purified recombinant SiaBb3 efficiently converted GM2 ganglioside [GalNAcβ1–4(Neu5Acα2-3)Galβ1–4Glcβ1-ceramide], sharing the same terminal trisaccharide structure with the Sd^a^ antigen, to lactosylceramide by releasing Neu5Ac and GalNAc in the presence of 0.1% sodium cholate. Hydrolysis of the GM2 oligosaccharide proceeds with the initial release of Neu5Ac, followed by the liberation of GalNAc, which was revealed by monitoring the reactions performed using catalytically inactive mutants for each domain of SiaBb3 and by analyzing the reactions of WT SiaBb3 on fluorescence-labeled oligosaccharides. Notably, the order of hydrolysis was reversed compared with that employed by mammalian lysosomal enzymes for GM2 degradation. Comparative *O*-glycomic analysis using fecal mucin as a substrate unequivocally demonstrated that SiaBb3 targets the Sd^a^ antigen of mucin *O*-glycans. The GH33-inactive SiaBb3 mutant retained Sd^a^ antigen–containing *O*-glycans intact, indicating that initial hydrolysis of Neu5Ac is essential for the subsequent removal of GalNAc. Taken together, these results indicate that SiaBb3 is a bifunctional enzyme specialized for the complete degradation of Sd^a^ antigens in host mucins.

Mucins are viscous glycoproteins that contain a high density of *O*-linked glycans attached to Ser or Thr in a Pro-Thr-Ser–rich domain (1). Mucins secreted into the gastrointestinal tract form a thick gel layer, providing an environment that is either favorable or unfavorable for gut microbes. The primary gel-forming mucin secreted from the stomach is MUC5AC, which is mainly modified by *O*-glycans with core 1 (Galβ1–3GalNAcα1-Ser/Thr) and core 2 (Galβ1–3[GlcNAcβ1–6]GalNAcα1-Ser/Thr) structures. In contrast, the mucin secreted from epithelia of the small and large intestines is largely MUC2, which primarily contains core 3 (GlcNAcβ1–3GalNAcα1-Ser/Thr) and core 4 (GlcNAcβ1–3[GlcNAcβ1–6]GalNAcα1-Ser/Thr) structures in addition to core 2 (1, 2). These core structures are frequently elongated by galactosylation and *N*-acetylglucosaminilation to form type 1 (-3Galβ1–3GlcNAcβ1-) or type 2 (-3Galβ1–4GlcNAcβ1-) chains. In some cases, branched chains are also formed by *N*-acetylglucosaminilation *via* β1–6 linkages. The nonreducing ends of *O*-glycans are frequently modified by various blood group antigens, including ABH and Lewis antigens, or by negatively charged saccharides, such as sialic acid and sulfation.

The Sd^a^ blood group antigen (GalNAcβ1–4[Neu5Acα2–3]Galβ1-*O*-R), also known as the CAD antigen, was initially identified as a determinant antigen of the Sid blood group system in red blood cells (3). The gene responsible for its biosynthesis, Sd^a^ β1,4-*N*-acetylgalactosaminyltransferase (*B4GALNT2*), is highly expressed in the colon, followed by the kidney, stomach, ileum, and rectum (4). Consistent with this expression pattern, the Sd^a^ antigen structure is abundant in the normal human colon, particularly in the distal regions, where it is attached to the *O*-glycans of the MUC2 mucin (5, 6, 7). Although the physiological functions of Sd^a^ antigens remain unclear, it has been suggested that synthesis of the Sd^a^ structure may mask the acceptor substrate (Neu5Acα2–3Gal) and thereby modulate host interactions with the microbiome (8). Supporting this notion, *B4GALNT2*-deficient mice display an altered gut microbiota characterized by reduced levels of *Helicobacter* spp. (9). Moreover, susceptibility to enteric pathogens, such as *Salmonella* and *Citrobacter*, is influenced by differences in *B4GALNT2* expression levels in mice (10, 11). Thus, Sd^a^ antigens are attracting increasing attention as key factors in host–microbe interactions; however, the mechanisms by which they influence the gut microbiome remain poorly understood.

The genus *Bifidobacterium* is a representative taxon that exerts beneficial effects on human health, as highlighted by its predominance in the infant gut microbiome through the assimilation of human milk oligosaccharides (12, 13) and its ability to produce the immunomodulatory compound indole-3-lactic acid *via de novo* synthesis or the indole-salvage pathway (14, 15, 16). Within the genus, *Bifidobacterium bifidum* is unique in its ability to degrade mucin *O*-glycans using an arsenal of cell surface–anchored glycoside hydrolases (GHs) (17, 18). Bacteria have evolved various GHs that act on mucin *O*-glycans to survive in highly competitive gut ecosystems. Particularly, the occurrence of α-sialidases, α-fucosidases, and sulfatases, which are indispensable for decapping the terminal modifications, has been documented for prominent commensal bacteria, including *Akkermansia*, *Bacteroides*, *Clostridium*, and *Ruminococcus* (19, 20, 21, 22). We have identified terminal glycan-degrading GHs from *B. bifidum*, including SiaBb1 and SiaBb2 α-sialidases (23, 24), BbhII 6-sulfo-β-*N*-acetylglucosaminidase (25), AgaBb α-galactosidase (26), AgnB α-*N*-acetylglucosaminidase (27), AfcA 1,2-α-fucosidase (28, 29), and AfcB 1,3/4-α-fucosidase (30). In addition, we have reported GHs capable of degrading internal chains and core structures, such as lacto-*N*-biosidase for type 1 chain (31), β-galactosidase (BbgIII) for type 2 chain (32), β-*N*-acetylglucosaminidase (BbhI) for β-1,3-linked GlcNAc (32, 33), β-*N*-acetylglucosaminidase (BbhIV) for β-1,6-linked GlcNAc (33), endo-α-*N*-acetylgalactosaminidase (EngBF) (34, 35), and exo-α-*N*-acetylgalactosaminidase (NagBb) (36) for mucin core structures.

In this study, we report the discovery of a unique bifunctional enzyme of *B. bifidum*, termed SiaBb3, which acts on Sd^a^ antigen structures. The enzyme possesses an α-sialidase domain, which shows a low similarity to the previously characterized GH33 α-sialidases, and a GH123 β-*N*-acetylgalactosaminidase (β-GalNAc-ase) domain in the same polypeptide. Using purified WT and mutant recombinant SiaBb3 enzymes, combined with mass spectrometry (MS)–based glycomic analysis, we showed how the enzyme degrades Sd^a^ antigens on mucin *O*-glycans and the same structure is present in the oligosaccharide of ganglioside GM2.

## Results

### Sequence analysis of SiaBb3

**Figure 1:**
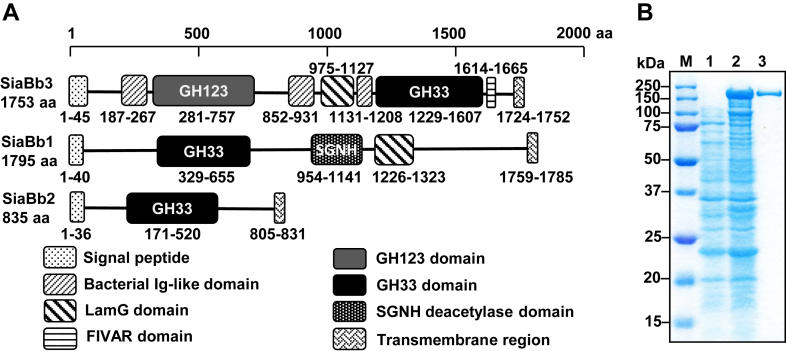
**Molecular cloning of SiaBb3.***A*, domain structures of SiaBb3, SiaBb1, and SiaBb2. *B*, SDS-PAGE of the recombinant SiaBb3. M, molecular size markers; lane 1, crude extract from *Escherichia coli* BL21(DE3)Δ*lacZ*-pET23b; lane 2, crude extract from *E. coli* BL21(DE3)Δ*lacZ*-pET23b/*siabb3*; lane 3, affinity-purified SiaBb3.

We previously characterized two α-sialidase genes, *siabb1* (23) and *siabb2* (24), from *B. bifidum* JCM 1254. The third α-sialidase gene was newly retrieved from the genome, which we named *siabb3* (locus tag BbifJCM1254_RS04480). The *siabb3* gene encodes a polypeptide of 1753 amino acid (aa) residues harboring a GH123 β-GalNAc-ase domain (aa 281–757) and a putative α-sialidase domain (aa residues 1229–1607), together with a signal peptide (aa 1–45), a class-E sortase-dependent cell wall anchoring motif [LIV][SA]XTG (aa 1725–1729) (37), and a membrane-spanning region (aa 1724–1752) (Fig. 1*A*). The amino acid sequence of the α-sialidase domain of SiaBb3, however, exhibited only 26% and 23% identity to those of SiaBb1 and SiaBb2, respectively. This low identity was reflected in the impaired Asp-box motif sequences (S/T-x-D-x-G-x-T/S-W/F/Y) of SiaBb3 (Fig. S1). Although SiaBb1 and SiaBb2 possessed five complete signature motifs, SiaBb3 contained only three. Moreover, while the orthologs of SiaBb1 and SiaBb2 were present across all strains of *B. bifidum* as well as in several strains of *Bifidobacterium longum* subsp. *longum* and *B. longum* subsp. *infantis*, SiaBb3 orthologs were found in only half of the *B. bifidum* strains (Table S1). The unusual sequence feature of the putative α-sialidase domain of SiaBb3 is also represented by the absence of BBPR_1596 (ADP36622.1, 1810 aa), the closest SiaBb3 homolog of *B. bifidum* PRL2010 (17), in the member list of GH33 in the CAZy database (as of March 2025) (38). BBPR_1596 is a member of GH123. The GH123 domain of SiaBb3 exhibits 36% and 33% identity to β-GalNAc-ases from *Clostridium perfringens* American Type Culture Collection 13124 (*Cp*Nga123) and from *Paenibacillus* sp. TS12 (NgaP), respectively (39, 40). Identity to the recently identified most phylogenetically distant β-GalNAc-ase from *B. longum* subsp. *infantis* JRPT (NagBl) is only 17% (41).

### Substrate specificities of the recombinant SiaBb3

Recombinant SiaBb3 (aa 46–1723) with a C-terminal His-tag was expressed in *Escherichia coli* and affinity purified. The purified preparation migrated as a single band of 180 kDa upon reduction by SDS-PAGE (Fig. 1*B*), which was in agreement with the molecular mass. The recombinant SiaBb3 hydrolyzed 4-methylumbelliferyl-α-d-Neu5Ac (4MU-α-Neu5Ac) and *p*-nitrophenyl (*p*NP)-β-GalNAc with turnover numbers of the enzyme for the substrates (0.2 mM) of 180 s^–1^ and 510 s^–1^, respectively. While a marginal activity was detected for *p*NP-β-GlcNAc (2.4 s^–1^), no activity was detected for other synthetic substrates examined, which included *p*NP-α-GalNAc, *p*NP-α-GlcNAc, *p*NP-α/β-Gal, *p*NP-α/β-Glc, *p*NP-α/β-Man, and *p*NP-α-Fuc. The α-sialidase activity of SiaBb3 toward 4MU-α-Neu5Ac was highest at pH 6.5 and 40 °C and was stable within the pH range of 4.5 to 10.0 and temperature below 40 °C. β-GalNAc-ase activity for *p*NP-β-GalNAc was optimal at pH 4.5 to 5.0 and 40 °C and was stable within the pH range of 4.0 to 8.0 and temperature below 50 °C. The α-sialidase activity was inhibited by 5 mM Zn^2+^ and Cu^2+^ ions, whereas β-GalNAc-ase activity was unaffected under the conditions, with only partial inhibition observed in the presence of Cu^2+^ ion. EDTA had no discernible effect on either of these activities.

To analyze the linkage specificity, we incubated SiaBb3 with 3′-sialyllactose (3′-SL), 6′-SL, and Neu5Acα2–8Neu5Ac, and quantified the released Neu5Ac by enzymatic colorimetric assay. The results revealed the preference of the enzyme for 6′-SL over 3′-SL, with their turnover numbers differing by 20-fold (11.7 *versus* 0.6 s^–1^). Hydrolysis of Neu5Acα2–8Neu5Ac was detectable by TLC analysis; however, the activity was too low to precisely determine the rate of hydrolysis.

### Action of SiaBb3 toward ganglioside GM2

As SiaBb3 exhibited dual α-sialidase and β-GalNAc-ase activities, we postulated its potential action on Sd^a^ antigens (GalNAcβ1–4[Neu5Acα2–3]Galβ1-*O*-R) present at the nonreducing ends of mucin *O*-glycans. We first employed GM2 ganglioside as the substrate to detect the activities because its oligosaccharide moiety shares the same structure as that of the Sd^a^ antigen. For comparison, purified SiaBb1 and SiaBb2 from *B. bifidum* and commercially available α-sialidases from *Arthrobacter ureafaciens*, *C. perfringens*, and *Vibrio cholerae* were also included in the analysis. While SiaBb1, SiaBb2, and SiaBb3 were each added at a concentration of 50 μg/ml (0.28, 0.64, and 0.27 μM, respectively), the other enzymes were added at 0.3 units/ml according to the manufacturer’s instructions.

**Figure 2:**
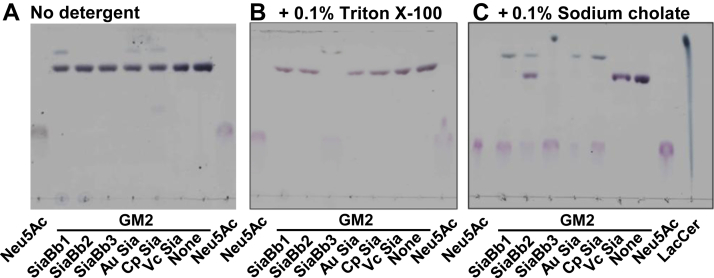
**Hydrolysis of GM2 by various α-sialidases.** GM2 (0.5 mg/ml) was incubated with α-sialidases (50 μg/ml for SiaBb1, SiaBb2, and SiaBb3; 0.3 units/ml for commercial α-sialidases) in the absence of any detergent (*A*), in the presence of 0.1% Triton X-100 (*B*) or 0.1% sodium cholate (*C*) in 50 mM sodium acetate buffer (pH 5.5) for 15 h. The reaction mixtures were analyzed by TLC. The experiment was performed three times, and one representative set of data is shown. Au, *Arthrobacter ureafaciens*; Cp, *Clostridium perfringens*; LacCer, lactosylceramide; Vc, *Vibrio cholerae*.

In the absence of detergent (Fig. 2, *left panel*), all α-sialidases tested showed a very limited activity on the Sd^a^ trisaccharide structure present in GM2 oligosaccharide. Faint spots with *R*_f_ values higher than that of GM2 were detected in the reaction mixture containing SiaBb1, *Arthrobacter*, and *Clostridium-*sialidases. Due to their amphipathic nature, glycosphingolipids are poor substrates for water-soluble glycosidases. In general, *in vitro* hydrolysis of sugar residues in glycosphingolipids by glycosidases requires the presence of a detergent (42). The addition of Triton X-100 enabled only SiaBb3 to hydrolyze the sugar chain of GM2, whereas the addition of sodium cholate, which might emulate physiological environments, enabled SiaBb3, SiaBb1, *Arthrobacter*- and *Clostridium-*sialidases to hydrolyze the substrate. Furthermore, a spot corresponding to lactosylceramide (LacCer) was produced upon incubation with SiaBb3, strongly suggesting the release of Neu5Ac and GalNAc from GM2.

### Degradation pathway of GM2 by SiaBb3

**Figure 3:**
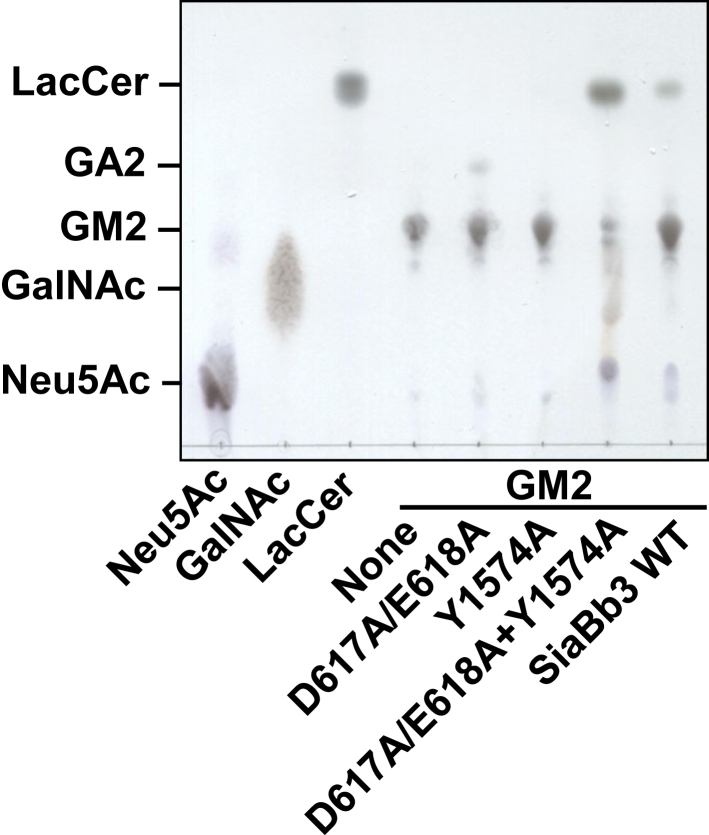
**Hydrolysis of GM2 by mutants of SiaBb3.** GM2 was incubated with either SiaBb3_D617A/D618A or SiaBb3_Y1574A, or both in the presence of 0.1% sodium cholate. The reaction mixtures were analyzed using TLC.

**Figure 4:**
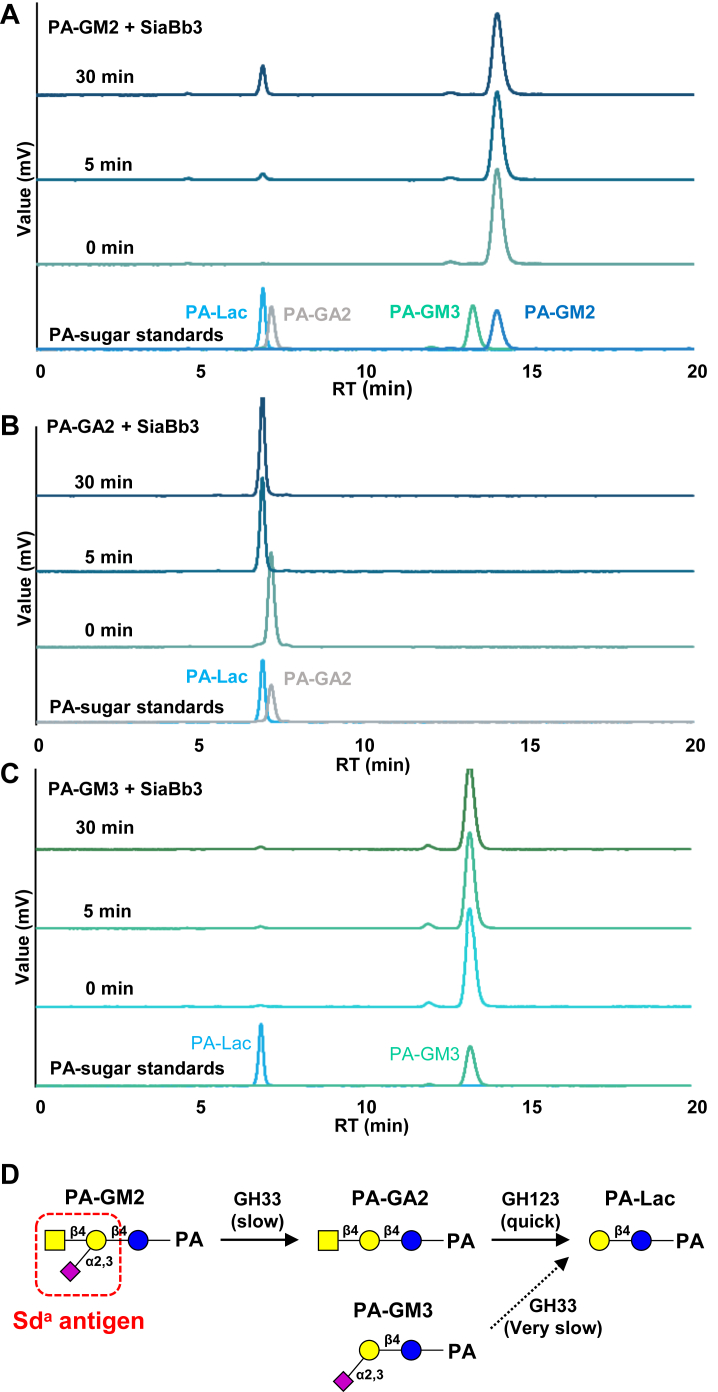
**Hydrolysis of fluorescently labeled GM2 oligosaccharide by SiaBb3_WT.***A*, degradation of PA–GM2 by SiaBb3. *B*, degradation of PA–GA2 by SiaBb3. *C*, degradation of PA–GM3 by SiaBb3. Reaction mixtures were analyzed by HPLC. *D*, proposed degradation pathways of GM2 by SiaBb3. The *dashed line* denotes a markedly reduced conversion rate. PA, pyridylamino.

To examine the mode of action of SiaBb3 on the Sd^a^ trisaccharide structure of GM2, we introduced loss-of-function mutation(s) in each catalytic domain. Regarding the GH123 domain, D617 and E618 were replaced with Ala because these residues were predicted to function as the catalytic proton donor and stabilizer of the oxazolium ion intermediate, respectively (39, 40). As expected, SiaBb3_ D617A/E618A completely lost its activity toward *p*NP-β-GalNAc. With respect to the α-sialidase domain, we individually introduced Ala substitutions into three Tyr residues (Y1241A, Y1558A, and Y1574A), as the sequence alignment did not clearly indicate a conserved catalytic residue. As a result, we found that Y1574A replacement leads to the complete loss of the activity toward 4MU-α-Neu5Ac. SiaBb3_D617A/E618A, which retains the α-sialidase activity, successfully degraded GM2 into asialo-GM2 (GA2). SiaBb3_Y1574A, which retains its β-GalNAc-ase activity, did not act on GM2. Coincubation of the two mutants with GM2 resulted in the formation of LacCer (Fig. 3). These results strongly suggest that the prior removal of α-Neu5Ac residue is indispensable for the following removal of β-GalNAc residue for SiaBb3 to degrade the Sd^a^ antigen structure on GM2. To further confirm this sequential mode of action, we used fluorescence-labeled GM2, GA2, and GM3 oligosaccharides as substrates and monitored their hydrolysis using HPLC (Fig. 4). When SiaBb3_WT was incubated with pyridylamino (PA)–GM2 oligosaccharide, a peak corresponding to PA–lactose appeared at 5 min, and its area increased up to 30 min. Notably, no peaks corresponding to PA–GA2 or PA–GM3 were detected throughout the incubation period (Fig. 4*A*). PA–GA2 was completely converted to PA–lactose within 5 min of incubation, whereas PA–GM3 was only marginally hydrolyzed after 30 min (Fig. 4, *B* and *C*). These results strongly suggest that the reaction catalyzed by SiaBb3 proceeds in two consecutive steps: the slow release of Neu5Ac from the GM2 oligosaccharide, followed by the rapid release of GalNAc from the GA2 oligosaccharide (Fig. 4*D*).

### SiaBb3 acts on Sd^a^ antigens of mucin

**Figure 5:**
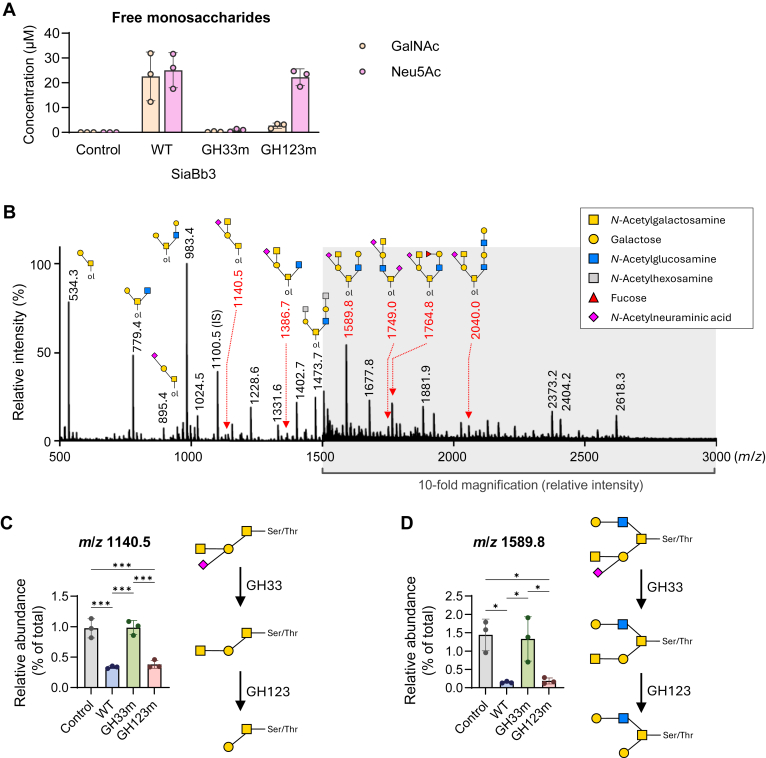
***O*-glycan analysis of SiaBb3-treated mouse fecal mucin.***A*, the amounts of the free GalNAc and Neu5Ac released after the incubation of the mouse fecal mucin sample with SiaBb3 variants, WT, SiaBb3_E1430A/Y1558A GH33 mutant (GH33m), and SiaBb3_D617A/E618A GH123 mutant (GH123m). Bars are shown as means ± SD of triplicates of independent experiments (*n* = 3). *B*, a full-mass spectrum of mouse fecal mucin *O*-glycans of untreated control (Table S2). The areas shaded in *gray* are shown at 10-fold magnified relative intensity. *Glycan cartoons* indicate the representative structures of each ion peak. “ol” indicates alditol. (*C*) and (*D*), bar plots of relative abundances (% of total) of each glycan ion at *m*/*z* 1140.5 and *m*/*z* 1589.8 with proposed degradation pathways of Sd^a^ antigen–containing *O*-glycans. Data are shown as means ± SD of triplicates of independent experiments (*n* = 3). Tukey’s multiple comparisons test was performed for statistical significance (∗∗∗*p* < 0.001; ∗*p* < 0.05).

Finally, we examined the effect of SiaBb3 on the Sd^a^ antigens present in mucin *O*-glycans using comparative glycomics. SiaBb3_WT, SiaBb3_ D617A/E618A mutant (GH123m), and SiaBb3_E1430A/Y1558A mutant (GH33m) were incubated with mucin extracted from the feces of germ-free mice. After obtaining the data in Figure 3, comparison with *C. perfringens* NanI sialidase (43) suggested that Y1558 is a likely nucleophilic residue and that E1430 may be involved in stabilizing Y1558. Therefore, we generated the SiaBb3_E1430A/Y1558A mutant and confirmed that its α-sialidase activity was completely abolished. During the first step of the analysis, we quantified the monosaccharides released into the supernatant after incubation (Fig. 5*A*). WT released free GalNAc and Neu5Ac to a similar extent, demonstrating the dual activity of this enzyme. GH123m also released Neu5Ac at a level similar to that of WT; however, its ability to release GalNAc was reduced to approximately one-tenth that of the WT enzyme (Fig. 5*A*). GH33m produces neither Neu5Ac nor GalNAc from mouse fecal mucin, which aligns with the above-mentioned finding that the removal of Neu5Ac is a prerequisite for the subsequent release of GalNAc during the hydrolysis of the Sd^a^ antigen trisaccharide of GM2 by SiaBb3.

*O*-Glycomics using MALDI–TOF/MS of untreated fecal mucin revealed 38 glycan ion peaks, indicating a relatively simple glycoform of fecal mucin from germ-free mice. These glycans mainly consisted of core 1 and core 2 structures, and modifications by fucosylation and sialylation appeared to be minimal, presumably because of the absence of gut bacteria that would otherwise induce the gene expression of glycosyltransferases in host cells (44). Nonetheless, *O*-glycans containing the Sd^a^ antigens were detected (Fig. 5*B*, Table S2). MS/MS fragment analysis suggested the presence of at least six glycan ions with the Sd^a^ antigen structure: *m*/*z* 1140.5, 1386.7, 1589.8, 1749.0, 1764.8, and 2040.0 (Table S2; Figs. S2, S3). Among them, the glycan ion peak at *m*/*z* 1140.5—which corresponds to a core 1 structure with an Sd^a^ antigen (Neu5Acα2-3[GalNAcβ1–4)Galβ1–3GalNAcα1-*O*-Ser/Thr) and its non-Sd^a^ isomers (Fig. S2*C*) showed a significant decrease in relative abundance upon treatment with either the WT or GH123m enzyme. No changes were observed in the glycan ion peaks after treatment with GH33m (Fig. 5*C*). The specific loss of the diagnostic fragment ions for the Sd^a^ antigen (*m*/*z* 606 and 865) in the MS/MS spectrum indicated degradation of the glycan structure (Fig. S2, *A* and *B*). These results revealed that, similarly to the sugar chain of GM2 ganglioside, Sd^a^-containing *O*-glycans are sequentially degraded: first by α-sialidase to yield GalNAcβ1–4Galβ1–3GalNAcα1-*O*-Ser/Thr and then by β-GalNAc-ase to produce Galβ1–3GalNAcα1-*O*-Ser/Thr (core 1) (Fig. 5*C*). A similar trend was observed for another glycan ion peak at *m*/*z* 1589.8 upon enzyme treatment (Fig. 5*D*; Fig. S3). In addition, SiaBb3 was also able to act on non-Sd^a^ sialylated structures, such as the sialylated core 1 at *m*/*z* 895.4. This finding suggests that the GalNAc moiety of the Sd^a^ antigen is not required for SiaBb3 to exert its α-sialidase activity.

## Discussion

The Sd^a^ antigen is expressed in the majority of humans as 97% of humans are positive (3). Its biosynthesis is catalyzed by BGALNT2, which modifies Neu5Acα2–3Galβ1–4(3)GlcNAcβ1-*O*-R that can otherwise serve as substrates of fucosyltransferases for the formation of tumor-associated sialyl Lewis x/a antigens (45, 46, 47). As sialyl Lewis x/a is also known to serve as ligands for the adhesion proteins of *Helicobacter pylori* (48, 49), it is possible that the host masks glycan antigens that would otherwise function as microbial binding ligands by converting those into Sd^a^-containing structures on the mucus layer, thereby regulating host–microbe interactions. On the other hand, mucin *O*-glycans generally serve as a nutrient source for gut microbes, and their glycan composition and structure are assumed to influence the formation of the microbiota. However, it has remained entirely unknown whether Sd^a^ structures can, in fact, be utilized by gut microbes.

In this study, we demonstrated that SiaBb3 from *B. bifidum* sequentially removes Neu5Ac and GalNAc from the GM2 oligosaccharide and Sd^a^ antigen of *O*-glycans. SiaBb3 contains both GH33 and GH123 enzymatic activities within a single polypeptide, appearing almost specialized for the degradation of Sd^a^ antigens, which strongly supports the notion that Sd^a^-containing mucin *O*-glycans can serve as a nutrient source for gut microbes. This degradation pathway is in contrast to that observed in mammalian lysosomes. Specifically, in mammalian lysosomes, the initial action on GM2 involves HEX-A β-hexosaminidase (GH20) that eliminates GalNAc from GM2 with the assistance of the GM2 activator protein, followed by NEU1 α-sialidase (GH33), removing Neu5Ac from GM3 to generate LacCer (42). Human NEU1 is unable to act on GM2, as evidenced by the accumulation of GM2 rather than GA2 in patients with Tay–Sachs disease (HEX-A deficiency). Despite the significant sequence dissimilarity of the SiaBb3 α-sialidase domain to the previously documented GH33 enzymes, including the human NEU1, it appears justifiable to retain it within the GH33 category as a derivative member. The α-sialidase domain of SiaBb3 may thus adopt an atypical folding to access the sterically densely packed Neu5Ac moiety of GM2 (50). SiaBb3 is the only enzyme that degrades GM2 in the presence of Triton X-100, a relatively gentle detergent commonly used to isolate glycosphingolipid- and cholesterol-rich lipid microdomains from cell membranes without destroying them (51). Conversely, SiaBb3 displayed a relatively weak activity toward synthetic substrates like 4MU-α-Neu5Ac. These findings suggest that SiaBb3 recognizes aglycon Gal at the (+1) subsite. The subsite accommodating the branched GalNAc may also be present; however, the binding could be weak, as Neu5Ac is also released from *O*-glycans without harboring Sd^a^ antigens. Detailed structural analysis revealed a distinctive substrate recognition mechanism employed by SiaBb3.

The occurrence of GH123 members is largely restricted to domain bacteria, and several GH123 enzymes have been biochemically characterized, such as NgaP from *Paenibacillus* sp. (40), *Cp*Nga123 from *C. perfringens* (39), and *Bv*GH123 from *Bacteroides vulgatus* (52). However, the range of GH123 has expanded recently, with representatives now reported in archaea, plants, and bacteria (41). In addition to enzymes that release GalNAc, those with 4-sulphated GalNAc- and the disaccharide (Galβ1–3GalNAc)-releasing activities have been identified. NgaP and SiaBb3 GH123 share the property of being able to act on GA2 but not on GM2. However, NgaP differs from SiaBb3 GH123 in that it acts on the terminal β1,3-linked GalNAc of Gb4 (GalNAcβ1–3Galα1–4Galβ1–4Glcβ1-ceramide) (Fig. S4). Similar to NgaP, *Cp*Nga123 degraded both GA2 and Gb4 oligosaccharides. *Bv*GH123 reportedly acts on both *p*NP-β-GalNAc and *p*NP-β-GlcNAc with a catalytic efficiency (*k*_cat_/*K*_*m*_) ratio of approximately 20:1 (52). SiaBb3 GH123 showed no activity on *p*NP-β-GlcNAc. Thus, the SiaBb3 GH123 domain might be characterized by its specificity for β1,4-linked GalNAc, which can differentiate it from previously reported GH123 enzymes. *B. bifidum* may have evolved SiaBb3 to degrade Sd^a^ antigens in the intestines. Because SiaBb3 did not act on ganglioside GM1 (Galβ1–3GalNAcβ1–4[Neu5Acα2–3]Galβ1–4GlcβCer), it likely lacks disaccharide-releasing activity (data not shown).

Occurrence of GHs capable of hydrolyzing β-GalNAc linkages is not limited to GH123 but includes GH20. β-*N*-Acetylhexosaminidase (GH20) from *Paenibacillus* sp. was reported to act on GA2 and Gb4 (53), suggesting its involvement in the degradation of GM2 oligosaccharide and Sd^a^ antigens in concert with other α-sialidases. Nonetheless, the discovery of SiaBb3, a bifunctional enzyme specialized in Sd^a^ antigen degradation, is intriguing when considering a previous study describing the association between the presence of mucin *O*-glycan degrading GHs and efficient mother–infant transmission of gut microbes during delivery (54). Thus, our findings warrant a study on *O*-glycan structural changes during pregnancy to better understand the coevolutionary relationship between *B. bifidum* and humans.

## Experimental procedures

### Bacterial strains and culture

Bifidobacterial strains were obtained from the Japan Collection of Microorganisms (JCM; RIKEN BioResource Center). Bacteria were cultured in GAM broth (Nissui Pharmaceutical) for 16 h at 37 °C under anaerobic conditions using an AnaeroPack (Mitsubishi Chemical).

### Cloning and expression of siabb3 in *E. coli*

Draft sequencing of the genome of *B. bifidum* JCM 1254 was performed using the Genome Sequencer 20 System (Roche Applied Science). The *siabb3* sequence was deposited in the DNA Data Bank of Japan under the accession number LC782750. More recently, Ojima *et al.* (13) determined the draft genome sequence of the strain JCM1254, in which the locus tag of *siabb3* was BbifJCM1254_RS04480. To construct the expression vector for SiaBb3, a DNA fragment encoding amino acids 46 to 1723 (without N-terminal signal peptide and C-terminal transmembrane region) was amplified by PrimeSTAR MAX DNA polymerase (Takara Bio) using genomic DNA from *B. bifidum* JCM 1254 as a template and the following primers: forward 5′-agagctcagtcgagcccgtgcagacc and reverse 5′-ataagcttgcccttcttgccggtatcg, digested with SacI and HindIII, and ligated into pET23b(+). The nucleotide sequence of the insertion in pET23b/*siabb3* was confirmed by Sanger sequencing. The *E. coli* BL21(DE3)Δ*lacZ* strain negative for β-galactosidase activity was transformed with pET23b/*siabb3* and cultured in Luria–Bertani liquid medium containing 100 μg/ml ampicillin at 25 °C until the absorbance at 600 nm reached 0.4. Then, to induce the expression, 0.5 mM isopropyl β-d-1-thiogalactopyranoside was added to the culture and incubated for 5 h at 25 °C. Cells were lysed by BugBuster Protein Extraction Reagent (Merck). After centrifugation, the supernatant was applied to a His GraviTrap (1 ml, GE Healthcare), and the adsorbed proteins were eluted using a stepwise imidazole concentration gradient in 50 mM sodium acetate buffer (pH 5.5) containing 250 mM NaCl. The active fractions were dialyzed against 50 mM sodium acetate buffer (pH 5.5). For SiaBb1 and SiaBb2, previously constructed vectors, pET23b/*siabb1* and pET23b/*siabb2*, were used, and the enzymes were purified in a similar manner (23, 24). Yields of the recombinant enzymes for SiaBb3_WT, SiaBb3_D617A/E618A (GH123m), SiaBb3_Y1574A, SiaBb3_E1430A/Y1558A (GH33m), SiaBb1, and SiaBb2 were 3.30, 2.95, 2.66, 2.41, 4.15, and 8.20 mg per 100 ml culture. Concentrations of the purified proteins were determined by using the theoretical absorption coefficient at 280 nm calculated based on the sequence (https://web.expasy.org/protparam/).

### Enzyme assay

*p*NP- and 4MU-monosaccharides, 3′-SL, 6′-SL, Gb4, α-sialidases from *C. perfringens* and *V. cholerae* were purchased from Sigma–Aldrich. GM2 was either a kind gift from Dr Yoshio Hirabayashi (Riken) or purchased from Matreya LLC. Neu5Acα2–8Neu5Ac and α-sialidase of *A. ureafaciens* were purchased from Nacalai Tesque. α-Sialidase activity was assessed using 4MU-α-Neu5Ac, whereas other glycosidase activities were determined using *p*NP substrates (23, 55) with 13.5 nM SiaBb3 in 50 mM sodium acetate buffer (pH 5.5) at 37 °C. Released Neu5Ac from sialyloligosaccharides (3′-SL, 6′-SL, and Neu5Acα2–8Neu5Ac) was measured by the colorimetric method using sialic acid aldolase (Toyobo) (56). To analyze the hydrolysis of glycosphingolipids, the reaction products were separated using silica gel TLC (Merck, 5553) with chloroform:methanol:0.02% CaCl_2_ (5:4:1, v/v/v) as the developing solvent and visualized using diphenylamine–aniline–phosphoric acid (57).

### Site-directed mutagenesis

Expression plasmids for the D617A/E618A, Y1241A, Y1558A, E1430A/Y1558A, and Y1574A mutants of SiaBb3 were generated by QuikChange site-directed mutagenesis using pET23b/*siabb3* as a template, and the mutations were confirmed using Sanger sequencing. Expression and purification of the mutant enzymes were carried out in the same manner as for WT SiaBb3.

### HPLC analysis

PA-oligosaccharides (50 pmol each; Takara Bio) were incubated with 56 nM SiaBb3 WT in 20 μl of 50 mM sodium acetate buffer (pH 5.5) at 37 °C. The reactions were quenched by heating at 95 °C for 5 min, and aliquots of the reaction mixtures were subjected to HPLC. Reversed-phase HPLC was performed using a Thermo U3000 system (Thermo Fisher Scientific) equipped with a Waters 2475 multi-λ fluorescence detector (Waters) and a TSK-gel ODS-80Ts column (4.6 × 250 mm; Tosoh) maintained at 40 °C. The mobile phase consisted of 100 mM acetate–triethylamine (pH 5.0) (solvent A) and 100 mM acetate–triethylamine (pH 5.0) containing 0.5% *n*-butanol (solvent B). Elution was performed at a flow rate of 0.8 ml/min using the following gradient program: 0% B (A/B = 100/0) for 0 to 8 min, a curved gradient (curve 8) to 30% B (0.15% *n*-butanol, A/B = 70/30) for 17 min, and equilibration at 0% B for 20 min. PA-oligosaccharides were detected at an excitation wavelength of 320 nm and an emission wavelength of 400 nm.

### SiaBb3 treatment of mucin

Fecal mucin samples extracted from germ-free mice (ICR males, 8 weeks old) prepared in our previous study (25) were treated with purified SiaBb3 enzyme as follows: 150 μg of mouse fecal mucin extract was incubated with 4.5 μM SiaBb3 in 150 μl of 50 mM sodium acetate buffer (pH 6.0) at 37 °C for 24 h. After enzymatic treatment, 300 μl of ice-cold acetone was added, and the mixture was kept at −30 °C overnight. The samples were then centrifuged to obtain protein pellets. The supernatants were subjected to high-performance anion-exchange chromatography–pulsed amperometric detection (HPAEC–PAD) analysis to quantify monosaccharides (described later), whereas protein pellets were used for *O*-glycan analysis.

### HPAEC–PAD analysis

Monosaccharides released from mouse fecal mucin upon incubation with SiaBb3 variants were quantified using HPAEC–PAD as described previously (25). A Dionex ICS-3000 system (Thermo Fisher Scientific) equipped with a CarboPac PA1 column (2 × 250 mm; Dionex) kept at 30 °C was used. Elution was performed at a flow rate of 0.25 ml/min using a solvent system consisting of water (mobile phase A), 250 mM NaOH (mobile phase B), and 1 M CH_3_COONa (mobile phase C). The elution scheme (% of mobile phase A/B/C) comprised 94/5.5/0.5 for the first 17 min to detect neutral sugars, including GalNAc, followed by a gradient of 46/50/4.0 for the next 10 min to detect Neu5Ac. The column was washed 35/50/15 for 10 min after each run. Standard curves were generated using the known sugar concentrations.

### O-Glycan analysis

*O*-glycan analysis was performed as previously described (25). Briefly, *O*-glycans from protein pellets (100 μg) were released by reductive β-elimination. Lacto-*N*-fucopentaose I (500 pmol; *m*/*z* 1100.5 [M + Na]^+^ after permethylation) was used as an external standard. The released glycan alditols and standard sugars were then purified and permethylated. Permethylated nonsulfated glycan alditols were dissolved in a 2,5-dihydroxybenzoic acid matrix solution (10 mg/ml in 50% methanol) and analyzed using MALDI-TOF/MS. Mass spectra were acquired on a neofleX instrument (Bruker Daltonics), operated in positive-ion reflector mode. Glycan composition was estimated from the monoisotopic *m*/*z* values using the GlycoMod tool (58). Only ion peaks with glycan compositions supported by MS/MS analysis in control samples and with mean abundances exceeding 10 pmol per 100 μg of mucin in at least one sample were considered.

## Data availability

Source data for this study are available from the corresponding authors upon reasonable request. Glycomics data were deposited in GlycoPOST (59) under the dataset identifier GPST000634.

## Ethical approval

This article does not contain any studies with animals performed by any of the authors.

## Supporting information

This article contains supporting information.

## Conflict of interest

The authors declare that they have no conflicts of interest with the contents of this article.
