# Supplementary material for: Unique bifunctional α-sialidase/β-N-acetylgalactosaminidase from Bifidobacterium bifidum acting on the Sda antigen
Source: J Biol Chem. 2025 Dec 30;302(2):111121. doi: 10.1016/j.jbc.2025.111121 (PMC12856340; doi:10.1016/j.jbc.2025.111121)
Supplement: Supporting Information [file mmc1.pdf]

Supplemental Table S1. Conservation of homologs of SiaBb1, SiaBb2 and SiaBb3 in various strains of *Bifidobacterium bifidum*, *B. longum* subsp. *longum* and *B. longum* subsp. *infantis*.

Species	Strain	SiaBb1	SiaBb2	SiaBb3
Bifidobacterium bifidum	JCM 1254	+	+	+
Bifidobacterium bifidum	JCM 1255 = ATCC 29521	+	+	+
Bifidobacterium bifidum	PRL2010	+	+	+
Bifidobacterium bifidum	BGN4	+	+	+
Bifidobacterium bifidum	LMG 13195	+	+	+
Bifidobacterium bifidum	S17	+	+	+
Bifidobacterium bifidum	DSM 20215	+	+	
Bifidobacterium bifidum	IPLA 20015	+	+	
Bifidobacterium bifidum	CECT 7366	+	+	
Bifidobacterium bifidum	NCIMB 41171	+	+	
Bifidobacterium bifidum	VIII-210	+	+	
Bifidobacterium bifidum	CAG:234		+	
Bifidobacterium longum subsp. *longum*	JCM 1217 = ATCC 15707	+	+	
Bifidobacterium longum	NCC2705	+	+	
Bifidobacterium longum	AGR2137	+	+	
Bifidobacterium longum	D2957	+	+	
Bifidobacterium longum	DJO10A	+	+	
Bifidobacterium longum	E18	+	+	
Bifidobacterium longum subsp. *infantis*	JCM 1222 = ATCC 15697	+	+	
Bifidobacterium longum subsp. *infantis*	CCUG 52486	+	+	
Bifidobacterium longum subsp. *infantis*	EK3	+	+	
Bifidobacterium longum subsp. *infantis*	157F	+	+	

+, Homolog is present.

Supplemental Table S2. Glycosyl compositions and glycan abundance of *O*-glycans from SiaBb3 variants-treated mouse fecal mucin.

m/*z*[a] (Observed)	*m*/*z* (Theoretical)	Glycosyl composition[b]	Diagnostic fragments of Sd[a] antigen in MS/MS (*m*/*z* 606, 865)	Relative abundance[c] (% of total)			
				Control	WT	GH33m	GH123m
	$[M+Na]^+$						
534.3	534.29	(Hex)1(HexNAc)1-ol		20.0 ± 3.0	21.5 ± 5.2	17.4 ± 4.1	19.3 ± 6.5
708.3	708.38	(dHex)1(Hex)1(HexNAc)1-ol		1.1 ± 0.1	1.2 ± 0.2	1.3 ± 0.4	1.3 ± 0.3
779.4	779.41	(Hex)1(HexNAc)2-ol		12.6 ± 1.0	13.6 ± 1.5	13.2 ± 1.9	14.1 ± 1.0
895.4	895.46	(NeuAc)1(Hex)1(HexNAc)1-ol		1.9 ± 0.2	0.6 ± 0.0	2.3 ± 0.6	0.6 ± 0.1
953.5	953.50	(dHex)1(Hex)1(HexNAc)2-ol		1.0 ± 0.1	1.3 ± 0.0	1.5 ± 0.4	1.3 ± 0.2
983.5	983.51	(Hex)2(HexNAc)2-ol		25.8 ± 2.1	26.2 ± 0.6	24.9 ± 1.0	25.4 ± 3.0
1024.5	1024.54	(Hex)1(HexNAc)3-ol		3.6 ± 0.1	3.8 ± 0.3	3.8 ± 0.1	4.7 ± 0.3
1069.5	1069.55	(NeuAc)1(dHex)1(Hex)1(HexNAc)1-ol		0.2 ± 0.0	0.3 ± 0.0	0.3 ± 0.1	0.3 ± 0.2
1140.5	1140.59	(NeuAc)1(Hex)1(HexNAc)2-ol	Yes	1.0 ± 0.1	0.3 ± 0.0	1.0 ± 0.1	0.4 ± 0.1
1157.6	1157.60	(dHex)1(Hex)2(HexNAc)2-ol		2.5 ± 0.3	3.3 ± 0.5	2.5 ± 0.2	2.7 ± 0.5
1187.6	1187.61	(Hex)3(HexNAc)2-ol		0.9 ± 0.1	1.3 ± 0.2	1.1 ± 0.1	1.1 ± 0.0
1199.6	1198.63	(dHex)1(Hex)1(HexNAc)3-ol		0.2 ± 0.0	0.3 ± 0.0	0.4 ± 0.1	0.3 ± 0.1
1228.6	1228.64	(Hex)2(HexNAc)3-ol		5.1 ± 0.6	5.5 ± 0.9	5.3 ± 0.6	6.3 ± 1.2
1256.6	1256.64	(NeuAc)2(Hex)1(HexNAc)1-ol		0.6 ± 0.0	0.2 ± 0.0	0.6 ± 0.1	0.2 ± 0.1
1269.6	1269.67	(Hex)1(HexNAc)4-ol		0.2 ± 0.0	0.3 ± 0.0	0.3 ± 0.1	0.3 ± 0.0
1331.6	1331.69	(dHex)2(Hex)2(HexNAc)2-ol		2.4 ± 0.4	2.2 ± 0.6	2.3 ± 0.4	2.1 ± 0.6
1344.6	1344.69	(NeuAc)1(Hex)2(HexNAc)2 -ol		0.8 ± 0.1	0.5 ± 0.1	0.9 ± 0.1	0.5 ± 0.0
1386.7	1385.71	(NeuAc)1(Hex)1(HexNAc)3-ol	Yes	0.7 ± 0.1	0.5 ± 0.1	1.0 ± 0.1	0.5 ± 0.1
1402.7	1402.73	(dHex)1(Hex)2(HexNAc)3-ol		5.7 ± 1.0	5.7 ± 1.5	5.8 ± 1.1	6.0 ± 1.7
1432.7	1432.74	(Hex)3(HexNAc)3-ol		1.4 ± 0.3	1.7 ± 0.4	1.5 ± 0.3	1.8 ± 0.2
1473.7	1473.77	(Hex)2(HexNAc)4-ol		6.1 ± 1.8	5.9 ± 1.3	6.3 ± 2.8	6.5 ± 1.1
1518.7	1518.78	(NeuAc)1(dHex)1(Hex)2(HexNAc)2-ol		0.5 ± 0.1	0.4 ± 0.1	0.6 ± 0.1	0.5 ± 0.1
1589.8	1589.81	(NeuAc)1(Hex)2(HexNAc)3-ol	Yes	1.4 ± 0.4	0.2 ± 0.0	1.3 ± 0.5	0.2 ± 0.1
1606.8	1606.83	(dHex)1(Hex)3(HexNAc)3-ol		0.3 ± 0.1	0.3 ± 0.0	0.3 ± 0.1	0.3 ± 0.0
1677.8	1677.87	(Hex)3(HexNAc)4-ol		0.6 ± 0.2	0.6 ± 0.1	0.6 ± 0.3	0.6 ± 0.2
1705.8	1705.86	(NeuAc)2(Hex)2(HexNAc)2-ol		0.1 ± 0.1	0.1 ± 0.0	0.2 ± 0.0	0.1 ± 0.0
1749.0	1746.89	(NeuAc)2(Hex)1(HexNAc)3-ol	Yes	0.2 ± 0.0	0.2 ± 0.1	0.2 ± 0.0	0.1 ± 0.0
1764.8	1763.90	(NeuAc)1(dHex)1(Hex)2(HexNAc)3-ol	Yes	0.5 ± 0.1	0.1 ± 0.0	0.5 ± 0.2	0.1 ± 0.0
1793.8	1793.91	(NeuAc)1(Hex)3(HexNAc)3-ol		0.2 ± 0.1	0.1 ± 0.0	0.3 ± 0.1	0.1 ± 0.0
1881.9	1881.97	(Hex)4 (HexNAc)4-ol		0.5 ± 0.2	0.5 ± 0.2	0.5 ± 0.2	0.5 ± 0.1
1923.9	1922.99	(Hex)3(HexNAc)5-ol		0.4 ± 0.2	0.3 ± 0.1	0.4 ± 0.2	0.4 ± 0.1
2026.0	2026.05	(dHex)2(Hex)3(HexNAc)4-ol		0.3 ± 0.1	0.3 ± 0.1	0.3 ± 0.1	0.2 ± 0.1
2040.0	2039.04	(NeuAc)1(Hex)3(HexNAc)4-ol	Yes	0.1 ± 0.0	0.1 ± 0.0	0.1 ± 0.0	0.1 ± 0.0
2127.0	2127.09	(Hex)4(HexNAc)5-ol		0.3 ± 0.1	0.2 ± 0.1	0.2 ± 0.2	0.3 ± 0.1
2169.0	2166.10	(Hex)3(HexNAc)6-ol		0.2 ± 0.2	0.2 ± 0.0	0.2 ± 0.2	0.2 ± 0.0
2373.2	2370.20	(Hex)4(HexNAc)6-ol		0.4 ± 0.3	0.2 ± 0.1	0.3 ± 0.3	0.3 ± 0.1
2404.2	2404.23	(dHex)3(Hex)4(HexNAc)4-ol		0.3 ± 0.2	0.3 ± 0.1	0.3 ± 0.1	0.2 ± 0.1
2618.3	2615.33	(Hex)4(HexNAc)7-ol		0.3 ± 0.3	0.1 ± 0.0	0.3 ± 0.2	0.1 ± 0.0
Total				100	100	100	100

a, The glycan ion peaks whose estimated amounts are larger than 10 pmol/100 ug fecal extracts in at least one sample and whose glycosyl compositions were confirmed by MS/MS analysis were shown.
b, Abbreviations for monosaccharides are as follows: dHex, deoxyhexose; Hex, hexose; HexNAc, *N*-acetylhexosamine; NeuAc, *N*-acetylneuraminic acid; -ol, alditol.
c, Data was shown in mean ± SD of three independent experiments (n = 3).

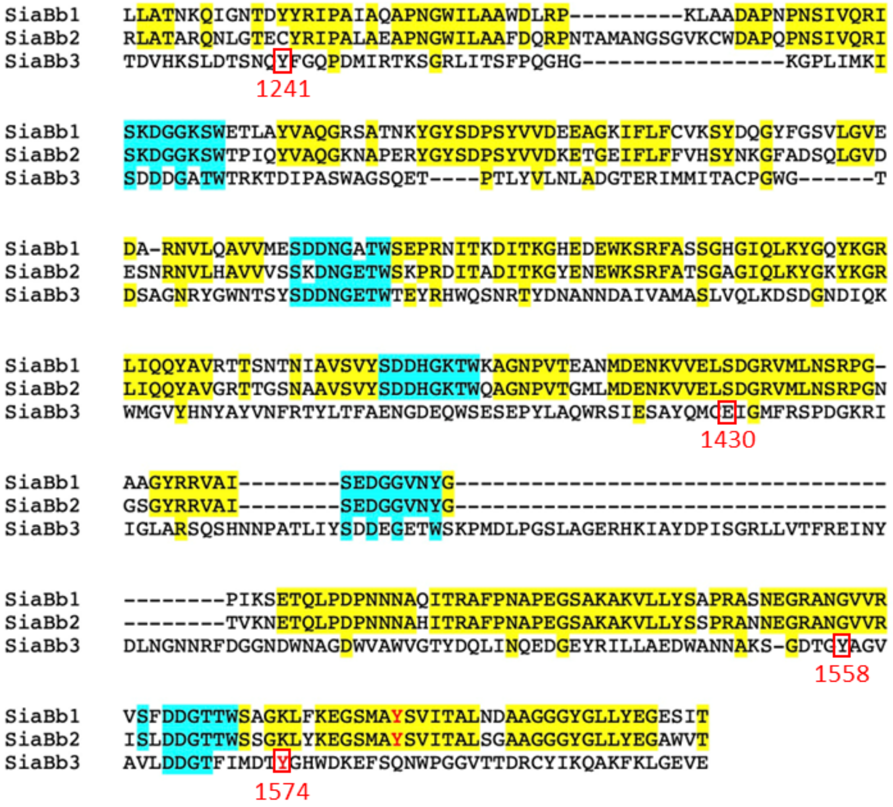

Supplemental Fig. S1. Sequence alignment of SiaBb1, SiaBb2 and SiaBb3.

Asp-box motif sequences (S/T-x-D-x-G-x-T/S-W/F/Y) are highlighted by light blue. Conserved residues are shown in yellow. Residues substituted with alanine in this study are indicated.

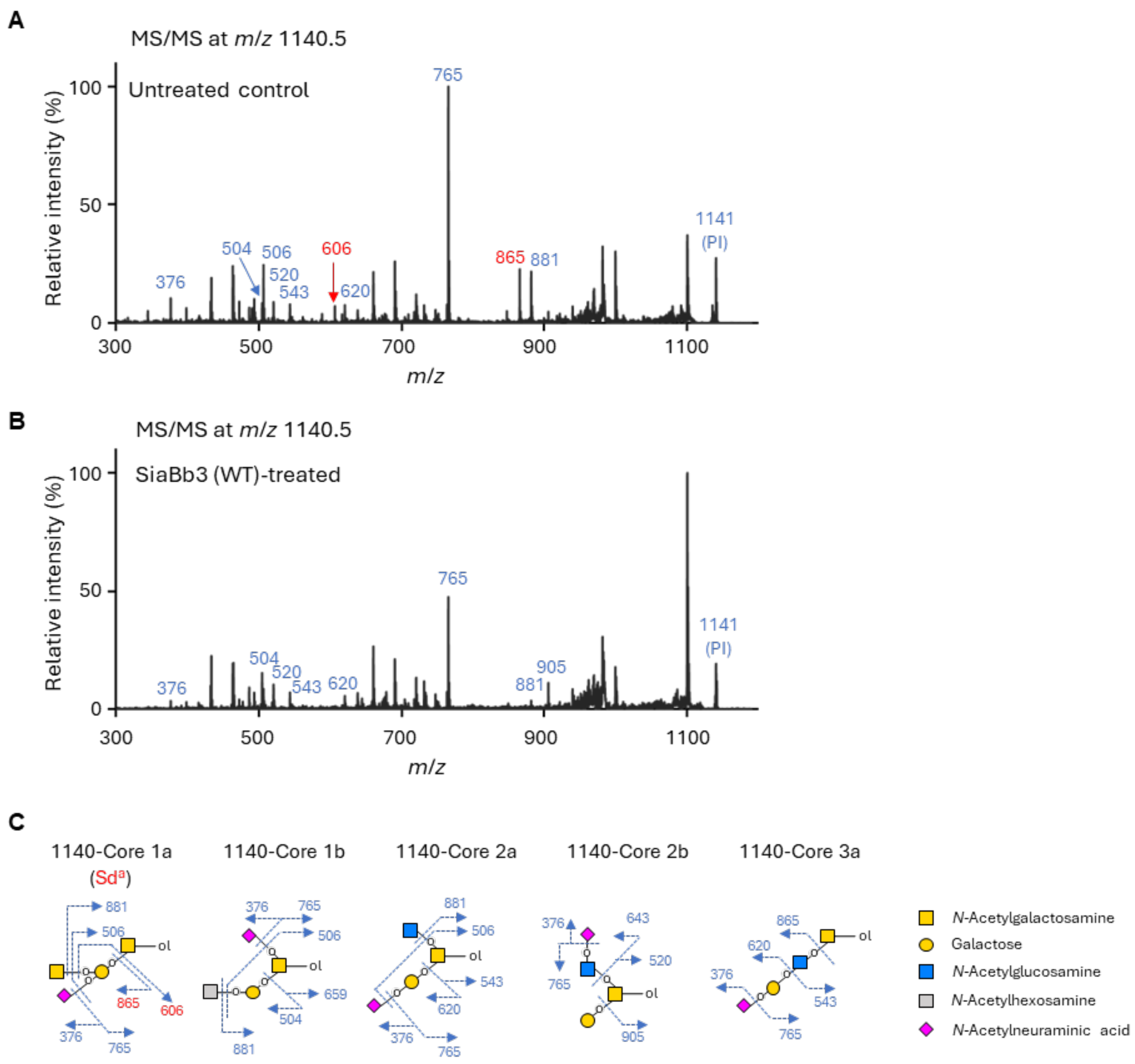

Supplemental Fig. S2. (**A**) MS/MS fragmentation pattern at *m/z* 1140.5 in the untreated control. (**B**) MS/MS fragmentation pattern at *m/z* 1140.5 in SiaBb3_WT-treated sample. Diagnostic fragments of Sd^a antigen (*m/z* 606 and 865) were lost. (**C**) Proposed glycan isomers at *m/z* 1140.5 in the untreated control, based on the MS/MS fragment ions.

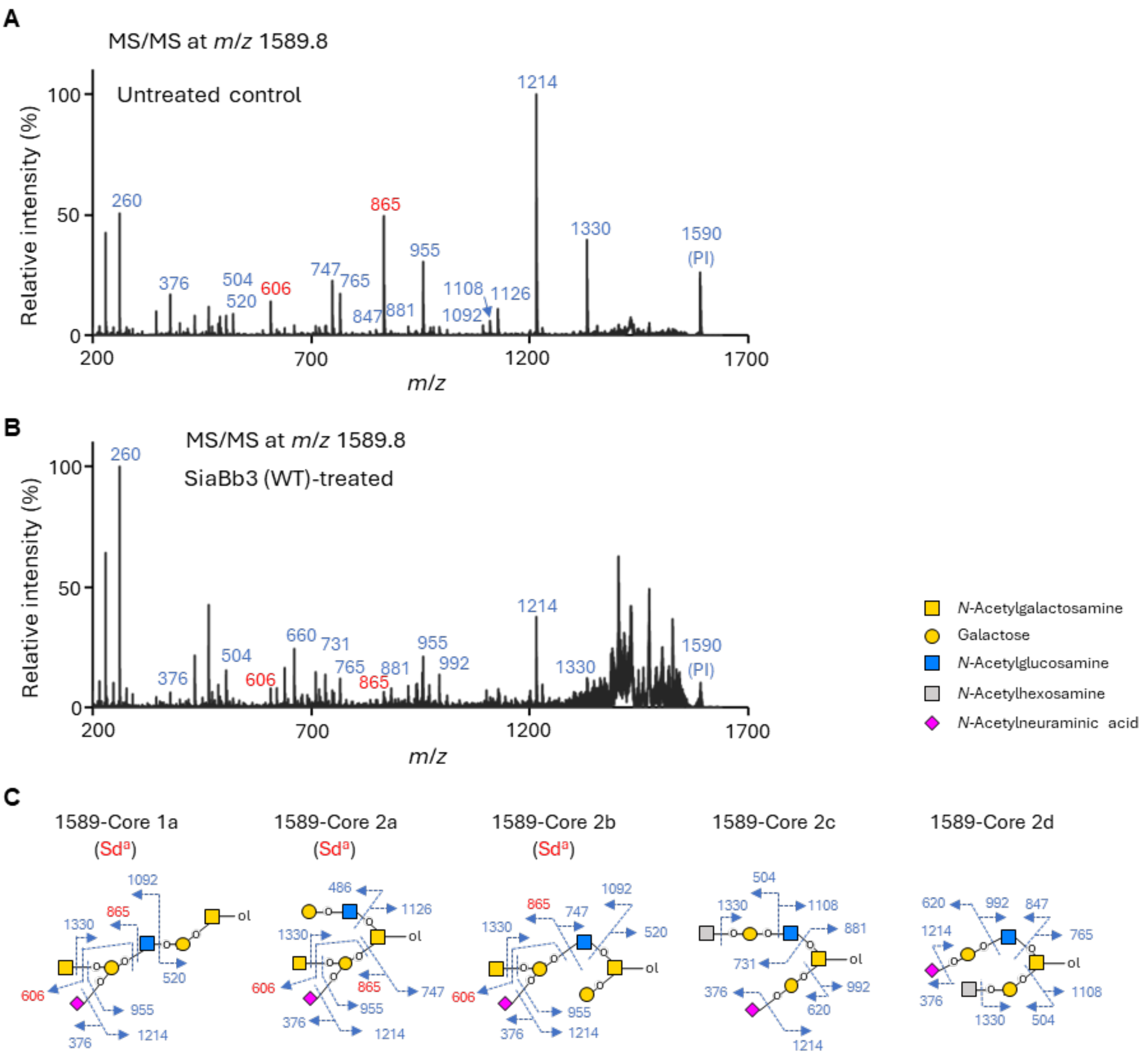

Supplemental Fig. S3. (**A**) MS/MS fragmentation pattern at *m*/*z* 1589.8 in the untreated control. (**B**) MS/MS fragmentation pattern at *m*/*z* 1589.8 in SiaBb3_WT-treated sample. Diagnostic fragments of Sd^{a} antigen (*m*/*z* 606 and 865) were relatively decreased. (**C**) Proposed glycan isomers at *m*/*z* 1589.8 in the untreated control, based on the MS/MS fragment ions.

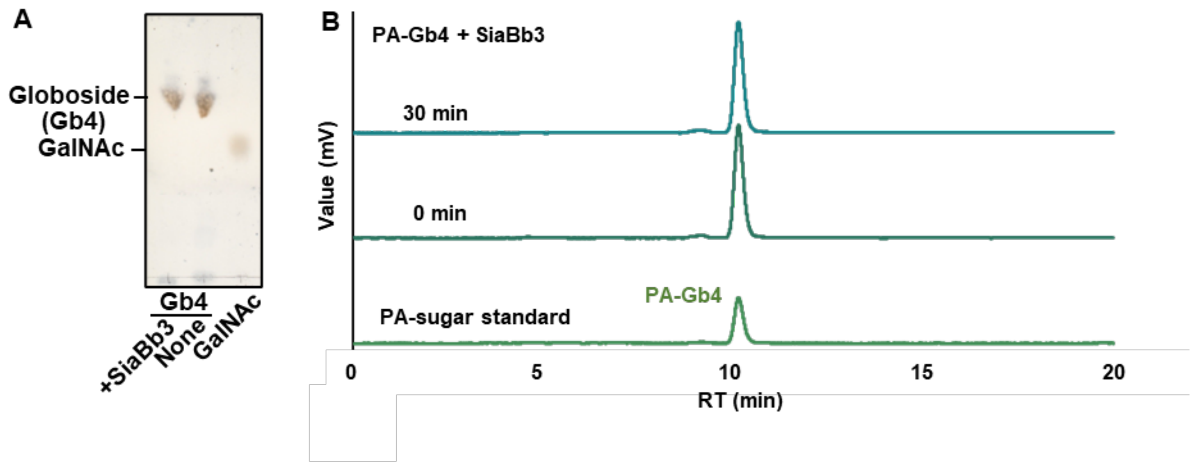

Supplemental Fig. S4. Hydrolysis of Gb4 and Gb4 oligosaccharide by SiaBb3. (**A**) Globoside (Gb4) was incubated with SiaBb3 and analyzed by TLC. (**B**) PA-Gb4 oligosaccharide was incubated with SiaBb3 and analyzed by HPLC.
